# [Benzyl(2-pyridylmeth­yl)amine]dichloridomercury(II)

**DOI:** 10.1107/S1600536808040695

**Published:** 2008-12-13

**Authors:** Hoe-Joo Seo, Young-Inn Kim, You-Soon Lee, Sung Kwon Kang

**Affiliations:** a Department of Chemistry, Pusan National University, Pusan 609-735, Republic of Korea; b Department of Chemistry Education and Center for Plastic Information Systems, Pusan National University, Pusan 609-735, Republic of Korea; c Department of Chemistry, Chungnam National University, Daejeon 305-764, Republic of Korea

## Abstract

The Hg atom in the title compound, [HgCl_2_(C_13_H_14_N_2_)], adopts a distorted tetra­hedral geometry, being ligated by two N atoms of the benzyl(2-pyridylmeth­yl)amine (bpma) ligand and two Cl atoms. The dihedral angle between the least-squares planes through the chelate ring and Cl—Hg—Cl atoms is 85.4 (1)°. The phenyl ring on the bpma ligand is twisted out of the pyridine plane, forming a dihedral angle of 76.0 (3)°. Disorder in this ring is also noted with two coplanar conformations having equal site occupancies.

## Related literature

For general background, see: Ojida *et al.* (2004 ▶). For background on luminescent mercury compounds, see: Yordanov & Roundhill (1998 ▶); Das *et al.* (2003 ▶); Haneline *et al.* (2002 ▶); Atoub *et al.* (2007 ▶). For related structures, see Kim *et al.* (2007 ▶, 2008 ▶).
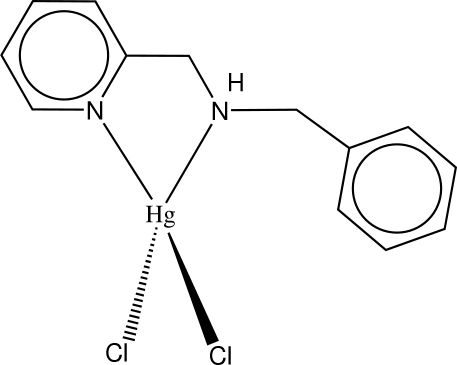


## Experimental

### 

#### Crystal data

[HgCl_2_(C_13_H_14_N_2_)]
*M* *_r_* = 469.75
Monoclinic, 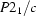

*a* = 13.1045 (3) Å
*b* = 13.8233 (3) Å
*c* = 8.3201 (2) Å
β = 91.135 (1)°
*V* = 1506.87 (6) Å^3^
*Z* = 4
Mo *K*α radiation
μ = 10.55 mm^−1^
*T* = 174 (2) K
0.12 × 0.11 × 0.10 mm

#### Data collection

Bruker SMART CCD area-detector diffractometer
Absorption correction: multi-scan (*SADABS*; Bruker, 2002 ▶) *T*_min_ = 0.290, *T*_max_ = 0.345
16218 measured reflections
3751 independent reflections
3258 reflections with *I* > 2σ(*I*)
*R*_int_ = 0.027

#### Refinement

*R*[*F* ^2^ > 2σ(*F* ^2^)] = 0.021
*wR*(*F* ^2^) = 0.045
*S* = 1.03
3751 reflections
195 parameters
H atoms treated by a mixture of independent and constrained refinement
Δρ_max_ = 0.69 e Å^−3^
Δρ_min_ = −0.82 e Å^−3^

### 

Data collection: *SMART* (Bruker, 2002 ▶); cell refinement: *SAINT* (Bruker, 2002 ▶); data reduction: *SAINT*; program(s) used to solve structure: *SHELXS97* (Sheldrick, 2008 ▶); program(s) used to refine structure: *SHELXL97* (Sheldrick, 2008 ▶); molecular graphics: *ORTEP-3 for Windows* (Farrugia, 1997 ▶); software used to prepare material for publication: *WinGX* (Farrugia, 1999 ▶).

## Supplementary Material

Crystal structure: contains datablocks global, I. DOI: 10.1107/S1600536808040695/tk2337sup1.cif

Structure factors: contains datablocks I. DOI: 10.1107/S1600536808040695/tk2337Isup2.hkl

Additional supplementary materials:  crystallographic information; 3D view; checkCIF report

## Figures and Tables

**Table 1:** Hydrogen-bond geometry (Å, °)

*D*—H⋯*A*	*D*—H	H⋯*A*	*D*⋯*A*	*D*—H⋯*A*
N8—H8⋯Cl2^i^	0.89 (4)	2.44 (4)	3.297 (3)	163 (3)

Symmetry code: (i) 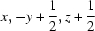.

**Fig. 1.**
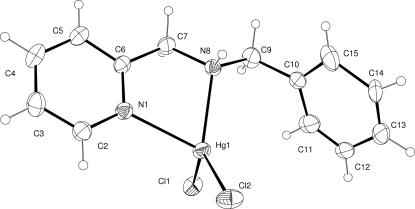
Molecular structure of (I), showing the atom-numbering scheme and 30% probability ellipsoids. For clarity, only one component of the disordered phenyl group is shown.

## supplementary crystallographic information

### Comment

Luminescent mercury compounds have attracted considerable attention because of the detection and extraction of the mercury (Yordanov & Roundhill, 1998; Das *et al.*, 2003) as well as the development of luminescent materials (Haneline *et al.*, 2002; Atoub *et al.*, 2007). Recently, we reported Zn(II) (Kim *et al.*, 2007) and Hg(II) (Kim *et al.*, 2008) compounds with bis(2-pyridylmethyl)amine and proposed these as blue fluorescent materials. As an extension of our study on luminescent chemosensors (Ojida *et al.*, 2004), herein, we report a Hg(II) chloride compound with *N*-benzyl-*N*-2-(pyridyl)methylamine (bpma), (I), and investigated its structural and luminescent properties.

In (I), Fig, 1, the Hg atom is ligated by two N atoms of the bpma ligand and two Cl atoms. The angles around Hg atom are in the range of 72.83 (9) - 123.37 (7)°, suggesting the coordination geometry around the Hg atom is best described as a distorted tetrahedron. The dihedral angle between the least-squares planes through N1—Hg1—N8 and Cl1—Hg1—Cl2 is 85.4 (1)°, which is close to 90° for a perfect tetrahedron. The phenyl ring on the bpma ligand is twisted out of the pyridine plane, and forms a dihedral angle of 76.0 (3)°. The major contacts in the crystal structure are N-H···Cl interactions and these combine to form a supramolecular chain, Table 1.

The free ligand (bpma) showed two strong blue (λ_max,PL_ = 379 and 449 nm in methylene chloride) fluorescent emissions upon 280 nm excitation, and Hg(bpma)Cl_2_ displayed an intense blue emission (λ_max,PL_ = 430 nm in dichloromethane) which is tentatively assigned to be an intraligand (IL) ^1^π-π^*^ transition.

### Experimental

All of the reagents and solvents were purchased from Aldrich and used without further purification. *N*-benzyl-*N*-(2-pyridylmethyl)amine (bpma) was synthesized from the reaction of 2-pyridinecarboxaldehyde, benzylamine and sodium borohydride. A solution benzylamine (20 mmol) in methanol (30 ml) was added slowly to a solution 2–2-pyridinecarboxaldehyde (20 mmol) in methanol (30 ml), and the mixture stirred for 3 h at room temperature. Sodium borohydride (20 mmol) in methanol (20 ml) was added and the solution was further stirred for 3 h at room temperature. The solution was evaporated to dryness and the residue extracted with dichloromethane to give bpma as yellow oil. To a stirred solution of mercuric chloride (10 mmol) in methanol (20 ml) was added bpma (10 mmol) in methanol (20 ml). The solution was stirred for 6 h at room temperature under a nitrogen atmosphere. The precipitates were filtered off and recrystallized from methanol to give (I) in a 61% yield. ^1^H-NMR for (I): (300 MHz, d_6_-DMSO) δ: 8.67 (d, 1H), 8.16 (t, 1H), 7.71 (d, 2H), 7.51 (m, 5H), 6.08 (s, 1H), 4.39(d, 2H), 4.12 (s, 2H).

### Refinement

The amine H8 atom was located in a difference map and refined freely with N—H = 0.89 (4) Å. The C-bound H atoms were included in the riding model approximation with C—H = 0.93–0.97 Å, and with *U*_iso_(H) = 1.2*U*_eq_(C). Disorder was noted in the structure and this modelled so that two sites were resolved for the phenyl-C12, C13, and C14 atoms. From refinement, each component of the disorder had a site occupancy factor = 0.50 (4).

### Crystal data

**Table:**

[HgCl_2_(C_13_H_14_N_2_)]	*F*(000) = 880
*M**_r_* = 469.75	*D*_x_ = 2.071 Mg m^−^^3^
Monoclinic, *P*2_1_/*c*	Mo *K*α radiation, λ = 0.71073 Å
Hall symbol: -P 2ybc	Cell parameters from 6766 reflections
*a* = 13.1045 (3) Å	θ = 2.9–28.3°
*b* = 13.8233 (3) Å	µ = 10.55 mm^−^^1^
*c* = 8.3201 (2) Å	*T* = 174 K
β = 91.135 (1)°	Block, colourless
*V* = 1506.87 (6) Å^3^	0.12 × 0.11 × 0.1 mm
*Z* = 4	

### Data collection

**Table:**

Bruker SMART CCD area-detector diffractometer	3258 reflections with *I* > 2σ(*I*)
φ and ω scans	*R*_int_ = 0.027
Absorption correction: multi-scan (*SADABS*; Bruker, 2002)	θ_max_ = 28.3°, θ_min_ = 1.6°
*T*_min_ = 0.290, *T*_max_ = 0.345	*h* = −15→17
16218 measured reflections	*k* = −18→18
3751 independent reflections	*l* = −11→11

### Refinement

**Table:**

Refinement on *F*^2^	0 restraints
Least-squares matrix: full	H atoms treated by a mixture of independent and constrained refinement
*R*[*F*^2^ > 2σ(*F*^2^)] = 0.021	*w* = 1/[σ^2^(*F*_o_^2^) + (0.0166*P*)^2^ + 1.085*P*] where *P* = (*F*_o_^2^ + 2*F*_c_^2^)/3
*wR*(*F*^2^) = 0.045	(Δ/σ)_max_ = 0.001
*S* = 1.03	Δρ_max_ = 0.69 e Å^−^^3^
3751 reflections	Δρ_min_ = −0.82 e Å^−^^3^
195 parameters	

### Special details

**Table:**

Geometry. All e.s.d.'s (except the e.s.d. in the dihedral angle between two l.s. planes) are estimated using the full covariance matrix. The cell e.s.d.'s are taken into account individually in the estimation of e.s.d.'s in distances, angles and torsion angles; correlations between e.s.d.'s in cell parameters are only used when they are defined by crystal symmetry. An approximate (isotropic) treatment of cell e.s.d.'s is used for estimating e.s.d.'s involving l.s. planes.

### Fractional atomic coordinates and isotropic or equivalent isotropic displacement parameters (Å^2^)

**Table:**

	*x*	*y*	*z*	*U*_iso_*/*U*_eq_	Occ. (<1)
Hg1	0.212834 (9)	0.445793 (9)	0.162371 (14)	0.03875 (5)	
N1	0.05042 (19)	0.39726 (18)	0.2591 (3)	0.0381 (6)	
C2	−0.0374 (3)	0.3898 (3)	0.1748 (4)	0.0468 (8)	
H2	−0.0373	0.4027	0.0651	0.056*	
C3	−0.1277 (3)	0.3639 (3)	0.2442 (5)	0.0550 (9)	
H3	−0.1876	0.3594	0.1829	0.066*	
C4	−0.1275 (3)	0.3447 (3)	0.4051 (5)	0.0540 (9)	
H4	−0.1878	0.3278	0.4551	0.065*	
C5	−0.0378 (3)	0.3505 (2)	0.4930 (4)	0.0462 (8)	
H5	−0.0365	0.3367	0.6024	0.055*	
C6	0.0507 (2)	0.3773 (2)	0.4161 (4)	0.0361 (6)	
C7	0.1496 (2)	0.3892 (3)	0.5091 (4)	0.0463 (8)	
H7A	0.1518	0.4535	0.5559	0.056*	
H7B	0.1517	0.3428	0.5966	0.056*	
N8	0.24046 (19)	0.37557 (19)	0.4098 (3)	0.0348 (5)	
H8	0.247 (3)	0.312 (3)	0.391 (4)	0.050 (10)*	
C9	0.3360 (3)	0.4089 (3)	0.4915 (4)	0.0526 (9)	
H9A	0.346	0.3736	0.5913	0.063*	
H9B	0.3302	0.4771	0.5173	0.063*	
C10	0.4254 (2)	0.3936 (3)	0.3868 (4)	0.0411 (7)	
C11	0.4612 (3)	0.4674 (3)	0.2916 (5)	0.0605 (11)	
H11	0.4251	0.5248	0.3025	0.073*	
C12	0.5321 (13)	0.473 (2)	0.1940 (19)	0.052 (4)	0.50 (4)
H12	0.5498	0.5298	0.1412	0.062*	0.50 (4)
C13	0.5821 (16)	0.384 (3)	0.173 (3)	0.066 (7)	0.50 (4)
H13	0.6361	0.381	0.1022	0.079*	0.50 (4)
C14	0.5540 (15)	0.304 (2)	0.252 (2)	0.065 (6)	0.50 (4)
H14	0.5862	0.2457	0.2295	0.078*	0.50 (4)
C12A	0.5511 (15)	0.4286 (18)	0.1850 (19)	0.053 (5)	0.50 (4)
H12A	0.5797	0.4699	0.1099	0.063*	0.50 (4)
C13A	0.5883 (19)	0.337 (2)	0.199 (4)	0.075 (7)	0.50 (4)
H13A	0.6418	0.3166	0.1356	0.09*	0.50 (4)
C14A	0.5477 (16)	0.2739 (17)	0.307 (4)	0.076 (6)	0.50 (4)
H14A	0.5745	0.2125	0.3251	0.092*	0.50 (4)
C15	0.4712 (3)	0.3051 (3)	0.3786 (6)	0.0694 (12)	
H15	0.4541	0.2522	0.4419	0.083*	
Cl1	0.22317 (7)	0.61872 (6)	0.18426 (10)	0.0500 (2)	
Cl2	0.23761 (8)	0.36292 (6)	−0.08585 (10)	0.0558 (2)	

### Atomic displacement parameters (Å^2^)

**Table:**

	*U*^11^	*U*^22^	*U*^33^	*U*^12^	*U*^13^	*U*^23^
Hg1	0.03980 (7)	0.04565 (7)	0.03086 (7)	−0.00518 (5)	0.00190 (5)	0.00120 (5)
N1	0.0373 (14)	0.0425 (14)	0.0344 (13)	−0.0046 (11)	0.0005 (11)	0.0004 (11)
C2	0.0426 (19)	0.058 (2)	0.0401 (18)	−0.0070 (16)	−0.0046 (15)	−0.0019 (16)
C3	0.0386 (19)	0.065 (2)	0.062 (2)	−0.0093 (16)	−0.0023 (17)	−0.0102 (19)
C4	0.044 (2)	0.055 (2)	0.064 (2)	−0.0122 (16)	0.0201 (17)	−0.0085 (18)
C5	0.048 (2)	0.0474 (18)	0.0439 (18)	−0.0041 (15)	0.0132 (15)	0.0000 (15)
C6	0.0383 (16)	0.0338 (14)	0.0365 (16)	0.0025 (13)	0.0069 (13)	−0.0041 (13)
C7	0.0431 (19)	0.068 (2)	0.0275 (16)	0.0032 (16)	0.0037 (13)	−0.0025 (15)
N8	0.0344 (13)	0.0390 (13)	0.0309 (13)	0.0012 (11)	−0.0024 (10)	−0.0015 (11)
C9	0.043 (2)	0.080 (2)	0.0349 (18)	−0.0055 (18)	−0.0100 (15)	−0.0113 (17)
C10	0.0325 (16)	0.062 (2)	0.0285 (15)	−0.0043 (14)	−0.0112 (12)	−0.0002 (14)
C11	0.046 (2)	0.085 (3)	0.050 (2)	−0.021 (2)	−0.0177 (18)	0.013 (2)
C12	0.031 (5)	0.083 (11)	0.040 (5)	−0.018 (7)	−0.014 (4)	0.018 (7)
C13	0.028 (7)	0.12 (2)	0.053 (6)	0.000 (11)	−0.008 (6)	0.011 (14)
C14	0.031 (8)	0.103 (18)	0.062 (9)	0.025 (10)	0.006 (6)	0.009 (8)
C12A	0.045 (11)	0.063 (14)	0.051 (6)	−0.013 (8)	−0.007 (8)	0.013 (10)
C13A	0.032 (8)	0.101 (18)	0.093 (18)	−0.006 (10)	0.013 (8)	−0.027 (13)
C14A	0.044 (7)	0.073 (9)	0.111 (18)	−0.005 (6)	−0.001 (10)	−0.003 (9)
C15	0.039 (2)	0.073 (3)	0.096 (3)	0.0058 (19)	−0.016 (2)	0.002 (2)
Cl1	0.0564 (5)	0.0400 (4)	0.0539 (5)	−0.0043 (4)	0.0104 (4)	−0.0042 (4)
Cl2	0.0875 (7)	0.0456 (4)	0.0346 (4)	0.0015 (4)	0.0060 (4)	−0.0064 (3)

### Geometric parameters (Å, °)

**Table:**

Hg1—N8	2.298 (2)	C9—H9A	0.97
Hg1—N1	2.387 (3)	C9—H9B	0.97
Hg1—Cl2	2.3895 (8)	C10—C15	1.365 (5)
Hg1—Cl1	2.4010 (8)	C10—C11	1.380 (5)
N1—C6	1.334 (4)	C11—C12	1.248 (17)
N1—C2	1.340 (4)	C11—C12A	1.58 (2)
C2—C3	1.375 (5)	C11—H11	0.93
C2—H2	0.93	C12—C13	1.403 (19)
C3—C4	1.365 (5)	C12—H12	0.93
C3—H3	0.93	C13—C14	1.35 (3)
C4—C5	1.375 (5)	C13—H13	0.93
C4—H4	0.93	C14—C15	1.524 (19)
C5—C6	1.386 (4)	C14—H14	0.93
C5—H5	0.93	C12A—C13A	1.37 (3)
C6—C7	1.506 (4)	C12A—H12A	0.93
C7—N8	1.475 (4)	C13A—C14A	1.36 (2)
C7—H7A	0.97	C13A—H13A	0.93
C7—H7B	0.97	C14A—C15	1.25 (2)
N8—C9	1.486 (4)	C14A—H14A	0.93
N8—H8	0.89 (4)	C15—H15	0.93
C9—C10	1.489 (5)		
			
N8—Hg1—N1	72.83 (9)	N8—C9—C10	110.7 (3)
N8—Hg1—Cl2	123.37 (7)	N8—C9—H9A	109.5
N1—Hg1—Cl2	107.11 (7)	C10—C9—H9A	109.5
N8—Hg1—Cl1	110.18 (7)	N8—C9—H9B	109.5
N1—Hg1—Cl1	107.67 (7)	C10—C9—H9B	109.5
Cl2—Hg1—Cl1	122.31 (3)	H9A—C9—H9B	108.1
C6—N1—C2	118.8 (3)	C15—C10—C11	118.6 (4)
C6—N1—Hg1	113.8 (2)	C15—C10—C9	120.6 (4)
C2—N1—Hg1	127.4 (2)	C11—C10—C9	120.8 (4)
N1—C2—C3	122.5 (3)	C12—C11—C10	133.4 (14)
N1—C2—H2	118.7	C10—C11—C12A	109.8 (9)
C3—C2—H2	118.7	C12—C11—H11	113.3
C4—C3—C2	118.5 (3)	C10—C11—H11	113.3
C4—C3—H3	120.7	C11—C12—C13	112.4 (18)
C2—C3—H3	120.7	C11—C12—H12	123.8
C3—C4—C5	119.7 (3)	C13—C12—H12	123.8
C3—C4—H4	120.1	C14—C13—C12	122 (2)
C5—C4—H4	120.1	C14—C13—H13	119.1
C4—C5—C6	119.0 (3)	C12—C13—H13	119.1
C4—C5—H5	120.5	C13—C14—C15	122.2 (14)
C6—C5—H5	120.5	C13—C14—H14	118.9
N1—C6—C5	121.4 (3)	C15—C14—H14	118.9
N1—C6—C7	117.8 (3)	C13A—C12A—C11	122.2 (15)
C5—C6—C7	120.8 (3)	C13A—C12A—H12A	118.9
N8—C7—C6	113.2 (2)	C11—C12A—H12A	118.9
N8—C7—H7A	108.9	C14A—C13A—C12A	120 (2)
C6—C7—H7A	108.9	C14A—C13A—H13A	119.8
N8—C7—H7B	108.9	C12A—C13A—H13A	119.8
C6—C7—H7B	108.9	C15—C14A—C13A	115 (2)
H7A—C7—H7B	107.8	C15—C14A—H14A	122.5
C7—N8—C9	112.7 (2)	C13A—C14A—H14A	122.5
C7—N8—Hg1	109.47 (18)	C14A—C15—C10	133.5 (13)
C9—N8—Hg1	113.3 (2)	C10—C15—C14	111.4 (11)
C7—N8—H8	108 (2)	C14A—C15—H15	101.8
C9—N8—H8	107 (2)	C10—C15—H15	124.3
Hg1—N8—H8	106 (2)	C14—C15—H15	124.3

### Hydrogen-bond geometry (Å, °)

**Table:**

*D*—H···*A*	*D*—H	H···*A*	*D*···*A*	*D*—H···*A*
N8—H8···Cl2^i^	0.89 (4)	2.44 (4)	3.297 (3)	163 (3)

Symmetry codes: (i) *x*, −*y*+1/2, *z*+1/2.
